# Empyema of the Ureteral Stump. An Unusual Complication Following Nephrectomy

**DOI:** 10.1100/tsw.2010.45

**Published:** 2010-03-05

**Authors:** Apostolos P. Labanaris, Vahudin Zugor, Robert Smiszek, Reinhold Nützel, Reinhard Kühn

**Affiliations:** ^1^ Department of Urology, Martha Maria Medical Center, Nurnberg, Germany; ^2^ Department of Urology, St. Antonius Hospital, Gronau, Germany

**Keywords:** nephrectomy, ureteral stump, empyema, clinical symptoms, diagnosis, therapy

## Abstract

A ureteral stump, which is the segment of the ureter left in place after nephrectomy, may occasionally give rise to a pathologic process called ureteral stump syndrome, which is clinically interpreted as febrile urinary tract infections, lower quadrant pain, and hematuria. Empyema of the ureteral stump, which belongs to this syndrome, is an uncommon disease entity presenting with a reported incidence of 0.8–1%. We present a case of empyema of the ureteral stump in a female patient 5 years postnephrectomy for a nonfunctioning kidney, and discuss the clinical presentation, radiologic diagnosis, and therapeutic options of this uncommon disease entity.

## INTRODUCTION

The standard therapeutic management of a poorly functioning or nonfunctioning kidney is nephrectomy with proximal ureterectomy. A ureteral stump, which is the segment of the ureter that is left in place after nephrectomy, may occasionally give rise to various symptoms. Although these symptoms are often attributed to nonurologic conditions, they are usually caused by a pathologic process called ureteral stump syndrome, which is clinically interpreted as febrile urinary tract infections, lower quadrant pain, and hematuria[1]. Empyema of the ureteral stump, which belongs to this syndrome, is an uncommon disease entity presenting with a reported incidence of 0.8–1% in patients who have undergone nephrectomy[1,2].

We present a case of empyema of the ureteral stump in a female patient 5 years postnephrectomy for a nonfunctioning kidney, and discuss the clinical presentation, radiologic diagnosis, and therapeutic options of this uncommon disease entity.

## CASE REPORT

In January 2006, a 24-year-old female patient presented to our institution with fever, left lower abdominal pain, vomiting, and nausea. The abdomen was slightly distended and tender over the lower left region, without signs of peritoneal irritation. Peristalsis remained active and bowel function was normal. The laboratory values exhibited an elevated white blood count of 16/nl and CRP levels of 1.68 mg/dl. Urinalysis exhibited 100 leukocytes with negative nitrates. There was no evidence of microscopic hematuria and the urine culture was negative. In 2001, her left kidney was removed through a flank incision due to a hypoplastic kidney. She reported that the last year she had been diagnosed with recurrent episodes of urinary tract infections, which had been always treated with antibiotics. After nephrectomy took place, a routine cystoscopy or urethrocystoscopy never took place. We decided to perform urethrocystoscopy to evaluate the bladder and the left ureteral stump. The bladder and the left ureteral orifice did not exhibit any signs of infection or pathology, and there were no signs of vesicoureteral reflux present. A ureteral catheter was inserted into the left orifice in order to inject a contrast medium, but the catheter could not be inserted more than 3 cm. The ureteral stump did not exhibit any signs of dilatation (Fig. 1). Because of the atypical nature of the complaints, the negative urethrocystoscopy findings, and the time interval between nephrectomy and onset of symptoms, urological disease was not considered a cause for the patient's complaints. The cause was attributed to sigmoid diverticulitis and the patient was referred to the surgical department for further evaluation. A sigma-colonoscopy was performed, ruling out sigmoid diverticulitis or some kind of malignant process. Computed tomography was then performed, revealing an oval cystic mass of approximately 16 × 6 cm in the left retrovesical space adjacent to the bladder. The wall of the mass exhibited a contrast medium encasement and was suspected as ovarian cystadenoma, abscess, or empyema of the left ureteral stump (Fig. 2). Surgical exploration was performed and exhibited a dilated left ureteral stump of approximately 16 × 6 cm, which was eventually dissected in toto (Fig. 3). The pathological examination revealed an empyema of the left ureteral stump. Since the surgical procedure took place, there has been no recurrence of urinary tract infections or associated pathology.

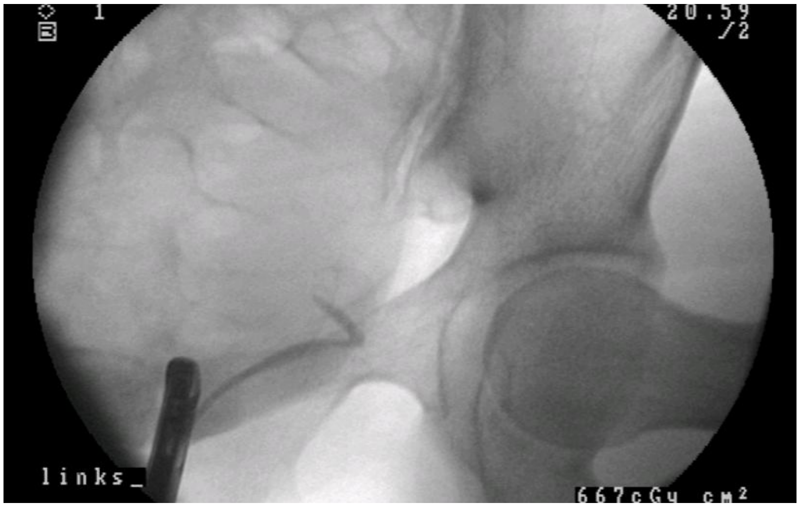

**FIGURE 1.** The ureteral catheter could not be inserted more than 3 cm into the left orifice.

## DISCUSSION

Excision of the entire ureter during nephrectomy for nonmalignant conditions in which the distal ureter has not somehow contributed to the pathogenesis of the underlying disease is not routinely performed. The reasons are that its removal would require either an extension of the incision, a second incision, or a transabdominal approach, and, furthermore, the fact that the retained ureteral stump is unlikely to become symptomatic if it was normal at the time of nephrectomy[2].

This rule however does not apply for all malignant conditions of the kidney. Although transitional cell carcinoma of the kidney is known to be a multifocal lesion and the standard practice to remove the entire ureter as well as a small cuff of bladder when performing nephrectomy for this disease has long

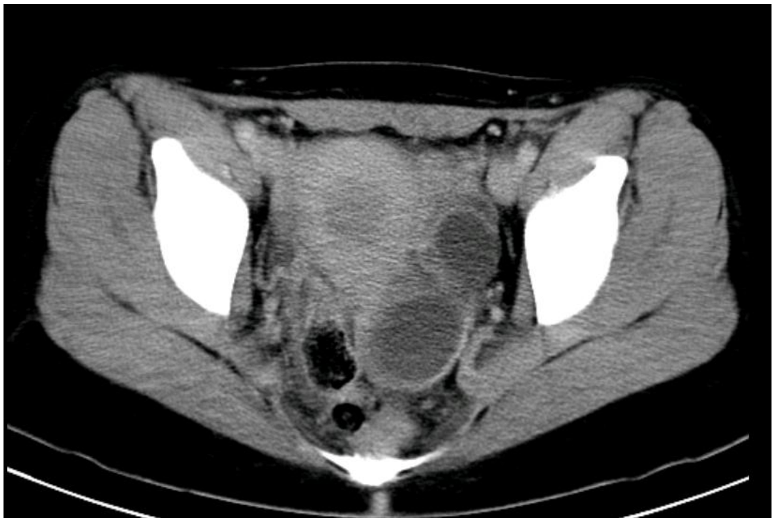

**FIGURE 2.** Computed tomography exhibiting an oval cystic mass of approximately 16 × 6 cm in the left retrovesical space adjacent to the bladder. The wall of the masses exhibited a contrast medium encasement.

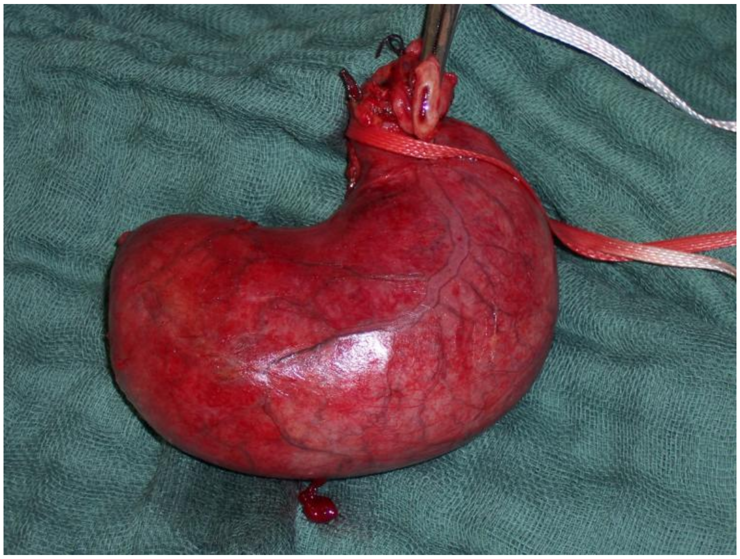

**FIGURE 3.** The pathologic specimen dissected in toto.

been established[3], renal cell carcinoma that metastasizes to the ureteral stump is rare[4]. There have been only a few series showing the natural history of the ureteral stump after nephrectomy. According to some investigators, the ureteral stump is a reservoir of urinary infections and should be removed by a dual approach with complete excision of the kidney and ureter[5,6]. Other investigators propose that only a long-remaining stump could be a risk factor in the pathogenesis of ureteric stump syndrome. Despite the view that the stump may retain some peristaltic activity, which prevents urinary stasis[7], a long-refluxing stump inevitably acts as a diverticulum from which urine cannot be effectively drained. Another significant observation is the role of dysfunctional voiding in causing acquired reflux into a long stump, leading to urinary stasis and infection[1]. However, investigators favoring subtotal ureterectomy consider that the distal ureteric stump is rarely a problem and elect to avoid the added morbidity of a second incision to remove the distal ureter[1,8,9].

If a ureteral stump eventually becomes symptomatic, then it is usually due to a pathologic process called ureteral stump syndrome, which is clinically interpreted as febrile urinary tract infections, lower quadrant pain, and hematuria[1]. Empyema of the ureteral stump, which belongs to this syndrome, is an uncommon disease entity presenting with a reported incidence of 0.8–1% in patients who have undergone nephrectomy[1,2].

There are many radiologic methods of imaging probable pathology of the ureteral stump. Of these, the most direct and definitive is retrograde ureterography[10]. If a ureteral orifice is still present at cystoscopy, a ureteral stump exists and must be excluded as a source of disease. However, if the ureteral orifice is stenotic or the stump cannot be catheterized, as seen in this patient, it can be evaluated by computed tomography, which is, however, not so sensitive and could lead to a false diagnosis.

The most popular treatment of empyema of the ureteral stump is the surgical resection of the stump, regardless of underlying diseases such as ectopic ureter, vesicoureteral reflux, or calculi in the stump[6]. However, some authors report transurethral fulguration as a safe and effective method for all patients with ureteral stump syndrome, as well those with as empyema of ectopic ureteral stump[11,12].

In summary, when postnephrectomy patients present with recurrent urinary tract infections and lower quadrant pain with or without hematuria, it is essential to rule out ureteral stump syndrome and, furthermore, ureteral stump empyema as a cause of these symptoms. Diagnosis should be suspected even when urethrocystoscopy findings are presumed negative.
